# Molecular Adaptation during Adaptive Radiation in the Hawaiian Endemic Genus *Schiedea*

**DOI:** 10.1371/journal.pone.0000008

**Published:** 2006-12-20

**Authors:** Maxim V. Kapralov, Dmitry A. Filatov

**Affiliations:** School of Biosciences, University of Birmingham Birmingham, United Kingdom; University of Cape Town, South Africa

## Abstract

**Background:**

“Explosive” adaptive radiations on islands remain one of the most puzzling evolutionary phenomena. The rate of phenotypic and ecological adaptations is extremely fast during such events, suggesting that many genes may be under fairly strong selection. However, no evidence for adaptation at the level of protein coding genes was found, so it has been suggested that selection may work mainly on regulatory elements. Here we report the first evidence that positive selection does operate at the level of protein coding genes during rapid adaptive radiations. We studied molecular adaptation in Hawaiian endemic plant genus *Schiedea* (*Caryophyllaceae*), which includes closely related species with a striking range of morphological and ecological forms, varying from rainforest vines to woody shrubs growing in desert-like conditions on cliffs. Given the remarkable difference in photosynthetic performance between *Schiedea* species from different habitats, we focused on the “photosynthetic” Rubisco enzyme, the efficiency of which is known to be a limiting step in plant photosynthesis.

**Results:**

We demonstrate that the chloroplast *rbcL* gene, encoding the large subunit of Rubisco enzyme, evolved under strong positive selection in *Schiedea*. Adaptive amino acid changes occurred in functionally important regions of Rubisco that interact with Rubisco activase, a chaperone which promotes and maintains the catalytic activity of Rubisco. Interestingly, positive selection acting on the *rbcL* might have caused favorable cytotypes to spread across several *Schiedea* species.

**Significance:**

We report the first evidence for adaptive changes at the DNA and protein sequence level that may have been associated with the evolution of photosynthetic performance and colonization of new habitats during a recent adaptive radiation in an island plant genus. This illustrates how small changes at the molecular level may change ecological species performance and helps us to understand the molecular bases of extremely fast rate of adaptation during island adaptive radiations.

## Introduction

The most dramatic “bursts” of adaptive radiation often occur within confined geographical regions (e.g. oceanic islands or inland freshwater lakes; e.g. [1]). Although island adaptive radiations may be viewed as extreme examples of evolutionary diversification, it is thought that major adaptive radiations in the history of our planet have been following the same evolutionary processes as island endemic radiations. Thus, islands may be viewed as evolutionary laboratories one can use to understand general processes of adaptation and speciation [1], [2].

“Explosive” island adaptive radiations are accompanied by tremendous phenotypical and ecological changes that suggest many genes might be under fairly strong positive selection. However, we do not know how natural selection works at the molecular level during adaptive radiation events. Island habitats are always limited in area, so island populations are limited in size. In addition, many island species are thought to evolve via colonization of new islands or habitats (“island hopping speciation”, [3]). Such colonization events should lead to a drastic reduction in population size. As the efficacy of selection is proportional to the product of the selective coefficient and the effective population size [4], the relatively small effective population size of island species should result in a reduced efficacy of natural selection. In small populations (e.g. in endemic island species), the dynamics of non-synonymous mutations is dominated by drift and the fixation probabilities of deleterious and advantageous mutations are expected to be approximately equal [5]. Few studies have investigated the action of selection at the molecular level during island adaptive radiations [6], [7]. These studies indicated some increase in non-synonymous (d*N*) to synonymous (d*S*) substitution rates in the Hawaiian silversword alliance, which may reflect slight relaxation of purifying selection in small island populations, but no convincing evidence of positive selection has been reported.

The small size of island populations may also limit the genetic variability required for natural selection to work, so the fast rate of phenotypic diversification on islands is quite surprising. Interspecific hybridization may be a possible source of additional genetic variation within species [2], [8], [9]. Closely related species are often cross-compatible and there are numerous examples of interspecific hybridization in plants and animals [10]. Even with low rates of introgression positively selected alleles can spread across several species [11]. Occasional events of interspecific hybridization allow adaptive radiations to be considered as metapopulations, where adaptive mutations may spread across several species significantly accelerating the adaptation process [12]. However, it is not known how common such sharing of adaptive mutations by several species might be.

In this paper we report the analysis of positive selection at the molecular level and the spread of adaptive alleles across several species in Hawaiian endemic plant genus *Schiedea* (*Caryophyllaceae*), which represents one of the largest plant adaptive radiations on Hawaii. *Schiedea* comprises 32 living (and at least two extinct) species adapted to a wide range of habitats from wet rainforest to dry desert-like conditions of coastal cliffs [13]. Among *Schiedea*'s most prominent evolutionary transitions have been remarkable changes in its growth habit, ranging from rainforest vines and perennial herbs through mesic and dry forest subshrubs and shrubs to cliff-dwelling shrubs [13] (see Table S1). The latter are particularly notable for a lineage within the *Caryophyllaceae* family which contains mainly herbaceous annuals and perennials.

*Schiedea* species from contrasting environments (e.g. rainforest vs. coastal cliffs) are dramatically different from each other in many physiological traits [13]. In particular, there are substantial differences in photosynthetic performance in *Schiedea*, suggesting that some of the protein coding genes involved in photosynthesis could be under positive selection. This motivated us to choose two chloroplast “photosynthetic” genes, *psbA* and *rbcL*, for phylogeny-based analysis of positive selection. The first gene, *psbA*, encodes photosystem II reaction center protein D1. Photosystem II is the first link in the chain of photosynthesis, it captures photons and uses the energy to extract electrons from water molecules [14]. The second gene, *rbcL*, encodes large subunits of Ribulose-1,5-bisphospate (RuBP) carboxylase/oxigenase (Rubisco; EC 4.1.1.39) which catalyzes the first step in net photosynthetic CO_2_ assimilation and photorespiratory carbon oxidation [15]. “The most abundant protein in the world”, Rubisco comprises about 40–50% of all soluble proteins in green plant tissues and is responsible for almost all carbon fixation on Earth. Despite its critical importance for life on our planet, this protein is notoriously inefficient in its function, creating a bottleneck, which limits plant growth [15]. This makes *rbcL* a likely target of positive selection, as any improvements in its function may drastically change plant growth rate. For comparison we also studied a non-photosyntetic chloroplast gene, *matK*, that encodes a protein of unknown function which is hypothesized to be involved in splicing in the chloroplast genome [16], [17].

Below we demonstrate that one of the studied photosynthetic genes, *rbcL*, evolved under positive selection during adaptive radiation in *Schiedea*. The differences in amino acid sequence among different *Schiedea* species could possibly account for the observed differences in photosynthetic performance and may have helped the genus to colonise a new habitat–dry sunny slopes and cliffs. Interestingly, the positive selection at *Schiedea rbcL* may have caused adaptive chloroplast haplotypes to spread across several *Schiedea* species, which are known to occasionally form hybrids in the wild [13]. This supports the view that sharing of adaptive mutations by several species may play a significant role in plant adaptive evolution [2], [9], [12].

## Results

### Positive Selection in *Schiedea rbcL*

**Figure 1:**
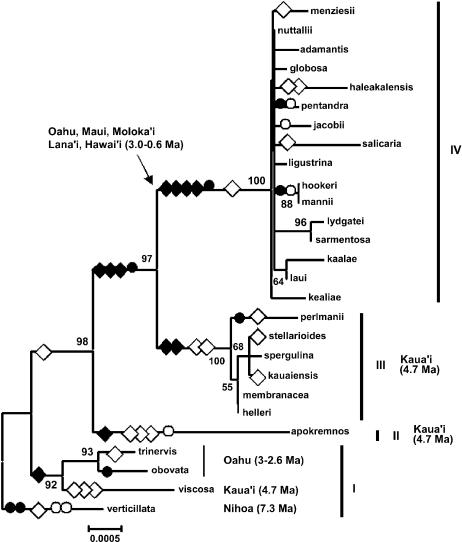
Neighbor joining tree of 27 *Schiedea* species constructed using three coding and four non-coding cpDNA regions. Numbers above branches are bootstrap support values (%). Non-synonymous (diamonds) and synonymous (circles) substitutions are shown for *rbcL* (black filled symbols) and *matK* (white symbols). The four clades are marked by Roman numbers.

Phylogenetic maximum likelihood analysis of selection at the molecular level assumes that the phylogeny of an analyzed gene is correct [18]. The published phylogeny of the genus *Schiedea* is based on morphology and ITS and ETS sequences and is relatively well established [13]. However, individual genes may have gene trees that differ from the species tree due to horizontal gene transfer and lineage sorting [2], [19], [20]. Thus, in order to conduct phylogenetic maximum likelihood analysis of selection in chloroplast genes it was essential to construct a robust gene tree of the *Schiedea* chloroplast DNA. For this purpose we sequenced fragments of *psbA, rbcL* and *matK* protein coding genes, as well as noncoding *trnL* intron, *psbA-trnK* and *trnL-trnF* intergenic spacers and *trnS-trnG* region (in total, 5.3 kb per individual, Table 1) from all 27 *Schiedea* species used in this study (Fig. 1 and supplementary table S1). As expected for a non-recombining chloroplast genome, the phylogenies based on individual chloroplast regions were consistent with each other, allowing us to concatenate the datasets, which resulted in a fairly well resolved phylogeny with high bootstrap support (25 haplotypes for concatenated dataset, Fig. 1). Interestingly, the *Schiedea* chloroplast gene tree (Fig. 1) substantially differed from the accepted ITS+ETS+morphology based phylogeny of the genus [13]. The plausible explanation for the observed discordance of the phylogenies as well as for rather “shallow” clades III and IV in the cpDNA tree (Fig. 1) could be the transition of cytotypes via interspecific hybridization and further fixation of favorable haplotypes within multiple species. Indeed, all the *Schiedea* species from cpDNA clade III live on the same island, Kaua'i, while the species from cpDNA clade IV inhabit several younger islands (O'ahu, Maui, Moloka'i, Lana'i, Hawai'i) that were connected to each other at various points in history of the archipelago. Thus, it seems likely that the cpDNA clades III and IV represent chloroplast hapoltypes that have spread across several species in Kaua'i, or younger islands. Strong positive selection in the *Schiedea* photosynthetic gene *rbcL* described below may have caused the cytotypes to spread across several species.

**Table 1:**
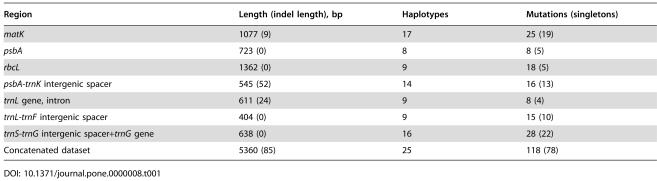
Investigated Chloroplast DNA Regions of 27 *Schiedea* Species

Region	Length (indel length), bp	Haplotypes	Mutations (singletons)
*matK*	1077 (9)	17	25 (19)
*psbA*	723 (0)	8	8 (5)
*rbcL*	1362 (0)	9	18 (5)
*psbA-trnK* intergenic spacer	545 (52)	14	16 (13)
*trnL* gene, intron	611 (24)	9	8 (4)
*trnL-trnF* intergenic spacer	404 (0)	9	15 (10)
*trnS-trnG* intergenic spacer+*trnG* gene	638 (0)	16	28 (22)
Concatenated dataset	5360 (85)	25	118 (78)

One out of the three investigated protein coding genes–*psbA*–appeared to be under strong purifying selection in *Schiedea*, showing no non-synonymous and only eight synonymous substitutions (Table 2). Moreover, when we compared *Schiedea*'s *psbA* with homologs from *Silene latifolia* (*Caryophyllaceae, Caryophyllales*; GenBank AB189069) and *Chenopodium rubrum* (*Chenopodiaceae, Caryophyllales*; GenBank Y14732) all 47 observed mutations again appeared to be synonymous.

**Table 2:**
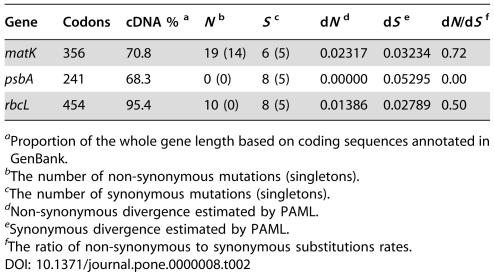
Non-synonymous and Synonymous Substitutions Rates in Three Investigated Chloroplast Genes of 27 *Schiedea* Species

Gene	Codons	cDNA % a	*N* b	*S* c	d*N* d	d*S* e	d*N*/d*S* f
*matK*	356	70.8	19 (14)	6 (5)	0.02317	0.03234	0.72
*psbA*	241	68.3	0 (0)	8 (5)	0.00000	0.05295	0.00
*rbcL*	454	95.4	10 (0)	8 (5)	0.01386	0.02789	0.50

a Proportion of the whole gene length based on coding sequences annotated in GenBank.

b The number of non-synonymous mutations (singletons).

c The number of synonymous mutations (singletons).

d Non-synonymous divergence estimated by PAML.

e Synonymous divergence estimated by PAML.

f The ratio of non-synonymous to synonymous substitutions rates.

Both *matK* and *rbcL* showed relatively high d*N*/d*S* averaged across the whole *Schiedea* phylogeny–0.72 and 0.50 respectively (Table 2). However, the distribution of non-synonymous substitutions on the phylogenetic tree for *rbcL* and all other regions was remarkably different (Table 2). While all ten non-synonymous substitutions in *rbcL* occurred in the internal branches, non-synonymous substitutions in *matK* as well as synonymous mutations in *rbcL, matK* and *psbA* and mutations in all non-coding regions appeared mainly in the terminal branches (Table 2; Fig 1). 2×2 contingency tests demonstrate that an unusually large number of amino acid replacements in the *Schiedea rbcL* occurred on the internal branches of the tree in comparison to synonymous mutations in *rbcL* as well as to non-synonymous and synonymous mutations in *matK* and *psbA* and mutations in non-coding regions (Table 3).

**Table 3:**
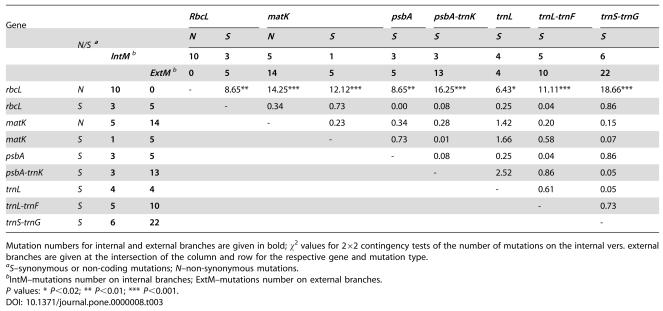
Comparisons of Mutation Numbers on Internal and External Branches of the *Schiedea* Chloroplast Gene Tree

**Gene**				*RbcL*		*matK*		*psbA*	*psbA-trnK*	*trnL*	*trnL-trnF*	*trnS-trnG*
	***N/S*** a			*N*	*S*	*N*	*S*	*S*	*S*	*S*	*S*	*S*
		***IntM*** b		**10**	**3**	**5**	**1**	**3**	**3**	**4**	**5**	**6**
			***ExtM*** b	**0**	**5**	**14**	**5**	**5**	**13**	**4**	**10**	**22**
*rbcL*	*N*	**10**	**0**	-	8.65**	14.25***	12.12***	8.65**	16.25***	6.43*	11.11***	18.66***
*rbcL*	*S*	**3**	**5**		-	0.34	0.73	0.00	0.08	0.25	0.04	0.86
*matK*	*N*	**5**	**14**			-	0.23	0.34	0.28	1.42	0.20	0.15
*matK*	*S*	**1**	**5**				-	0.73	0.01	1.66	0.58	0.07
*psbA*	*S*	**3**	**5**					-	0.08	0.25	0.04	0.86
*psbA-trnK*	*S*	**3**	**13**						-	2.52	0.86	0.05
*trnL*	*S*	**4**	**4**							-	0.61	0.05
*trnL-trnF*	*S*	**5**	**10**								-	0.73
*trnS-trnG*	*S*	**6**	**22**									-

Mutation numbers for internal and external branches are given in bold; χ^2^ values for 2×2 contingency tests of the number of mutations on the internal vers. external branches are given at the intersection of the column and row for the respective gene and mutation type.

a *S*–synonymous or non-coding mutations; *N*–non-synonymous mutations.

b IntM–mutations number on internal branches; ExtM–mutations number on external branches.

*P* values: * *P*<0.02; ** *P*<0.01; *** *P*<0.001.

To test for the presence of codons under positive selection in *matK* and *rbcL* we used likelihood ratio tests (LRTs) to compare the nested models allowing for variation in d*N*/d*S* ratio across codons [21]. In this analysis we compared the following pairs of models implemented in codeml program [18]: M1a/M2a [22], M7/M8 [21] and M8a/M8 [23]. Model M1a allows two classes of sites: one class with d*N*/d*S* varying freely between 0 and 1, and another one with d*N*/d*S* = 1 [22]. Model 2a has an additional class of sites, which can accommodate codons with d*N*/d*S*>1 [21]. The model M2a fits *rbcL* data significantly better than model M1a (χ^2^ = 12.88, *P* = 0.0016, df = 2), while there is no significant difference in fit of the two models to *matK* data (χ^2^ = 1.82, *P* = 0.4025, df = 2). In another nested pair of models M7 assumes that all codons have d*N*/d*S* distributed according to discrete beta distribution between 0 and 1, while model M8 allows for an additional class of codons with d*N*/d*S*>1 [21]. The comparison of these two models in a LRT is a test for the presence of a class of codons with d*N*/d*S*>1 [21]. The model M8 fits *rbcL* data significantly better than model M7 (χ^2^ = 13.25, *P* = 0.0013, df = 2), while there is no significant difference in fit of the two models for *matK* data (χ^2^ = 1.87, *P* = 0.3926, df = 2). Under the model M8 about 4% of codons in *rbcL* fall into the positively selected class, which had d*N*/d*S* = 13.92. A more stringent test for positive selection compares models M8 and M8a, which is the same as model M8, but the class of codons with d*N*/d*S*>1 in M8 is forced to have d*N*/d*S* = 1 in M8a. This LRT specifically tests whether the d*N*/d*S* for codons falling into this class is significantly larger than unity [23]. While in M8-M8a comparison there was no significant difference in fit for *matK* data (χ^2^ = 1.82, *P* = 0.1773, df = 1), application of this test to *Schiedea rbcL* demonstrated that this gene does have codons with d*N*/d*S* significantly larger than unity (χ^2^ = 12.87, *P* = 0.0003, df = 1), providing strong evidence for positive selection.

### Amino Acid Substitutions in *Schiedea RbcL*

The summary of the amino acid substitutions in *Schiedea rbcL* and their possible effects is presented in Table 4. Throughout the text amino acid positions are numbered according to spinach *rbcL* for which protein cristal structure is available [24]. Nine out of ten amino acid substitutions occurred on the branches of the phylogeny predating the split of the clades III and IV or leading to the clades III and IV (Fig. 1).

**Table 4:**
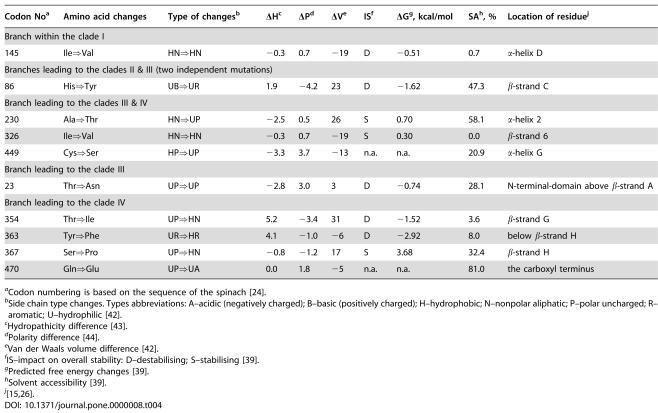
Physical Properties of Amino Acid Substitutions in *Schiedea*'s *RbcL*

Codon No^a^	Amino acid changes	Type of changes^b^	Δ</emph>H^c^	ΔP^d^	ΔV^e^	IS^f^	ΔG^g^, kcal/mol	SA^h^, %	Location of residue^j^
Branch within the clade I									
145	Ile⇒Val	HN⇒HN	−0.3	0.7	−19	D	−0.51	0.7	α-helix D
Branches leading to the clades II & III (two independent mutations)									
86	His⇒Tyr	UB⇒UR	1.9	−4.2	23	D	−1.62	47.3	β-strand C
Branch leading to the clades III & IV									
230	Ala⇒Thr	HN⇒UP	−2.5	0.5	26	S	0.70	58.1	α-helix 2
326	Ile⇒Val	HN⇒HN	−0.3	0.7	−19	S	0.30	0.0	β-strand 6
449	Cys⇒Ser	HP⇒UP	−3.3	3.7	−13	n.a.	n.a.	20.9	α-helix G
Branch leading to the clade III									
23	Thr⇒Asn	UP⇒UP	−2.8	3.0	3	D	−0.74	28.1	N-terminal-domain above β-strand A
Branch leading to the clade IV									
354	Thr⇒Ile	UP⇒HN	5.2	−3.4	31	D	−1.52	3.6	β-strand G
363	Tyr⇒Phe	UR⇒HR	4.1	−1.0	−6	D	−2.92	8.0	below β-strand H
367	Ser⇒Pro	UP⇒HN	−0.8	−1.2	17	S	3.68	32.4	β-strand H
470	Gln⇒Glu	UP⇒UA	0.0	1.8	−5	n.a.	n.a.	81.0	the carboxyl terminus

a Codon numbering is based on the sequence of the spinach [24].

b Side chain type changes. Types abbreviations: A–acidic (negatively charged); B–basic (positively charged); H–hydrophobic; N–nonpolar aliphatic; P–polar uncharged; R–aromatic; U–hydrophilic [42].

c Hydropathicity difference [43].

d Polarity difference [44].

e Van der Waals volume difference [42].

f IS–impact on overall stability: D–destabilising; S–stabilising [39].

g Predicted free energy changes [39].

h Solvent accessibility [39].

j [15], [26].

For four out of ten amino acid substitutions in *Schiedea rbcL* (residues 86, 230, 326 and 449) a Bayesian posterior probability of positive selection larger than 0.99 was shown by the Bayes Empirical Bayes analysis implemented in the PAML package [25]. Three of these residues (positions 230, 326 and 86) reside in regions that play key role in the functioning of Rubisco enzyme.

Replacement Ala230⇒Thr230 occurred on the branch leading to the clades III and IV of the *Schiedea* chloroplast gene tree (Fig. 1). Residue 230 interacts with the βA-βB loop of small subunit [26]. This residue 230 is highly solvent accessible (about 60% of total surface area; Table 4) and has a hydrogen bond with residue 10 of the small subunit of Rubisco. Replacement Ala230⇒Thr230 significantly decreases hydrophobicity of the residue that has a stabilizing effect in this position (Table 4).

Replacement Ile326⇒Val326 happened on the branch leading to the clades III and IV of the gene tree (Fig. 1). Residue 326 has six internal contacts and located inside of the protein molecule in the β-strand flanking loop 6, a flexible element that folds over substrate during catalysis and plays a key role in discriminating between CO_2_ and O_2_ in competing RuBP carboxylation and oxygenation reactions of Rubisco [15], [26]. Although Ile and Val have similar properties, Val is smaller and such replacement should increase the overall molecule stability (Table 4).

Interestingly, replacement His86⇒Tyr86 happened twice independently in *Schiedea* phylogeny–on the branches leading to the clade II and to the clade III (Fig. 1). Residue 86 is highly solvent accessible (about 50% of total surface area; Table 4) and may be a part of the Rubisco activase recognition region located in the N-terminal domain [15], [27]. The activase recognition region provides a physical contact between Rubisco and Rubisco activase, an ATP-dependent enzyme that releases tight-binding sugar phosphates from the Rubisco active site and facilitates conversion of Rubisco from the closed to the open conformation. Rubisco activase plays a vital role in the response of photosynthesis to temperature [27]. Properties of His and Tyr are very different: after His86⇒Tyr86 replacement hydrophobicy and volume of residue increased and polarity decreased dramatically making the molecule more tight while less soluble (Table 4). Although this is predicted to decrease stability of the molecule (Table 4), this analysis was done without taking interaction with Rubisco activase into account (for which no protein structure is available). Decreased polarity and increased hydrophobicy of the residue interacting with Rubisco activase may result in tighter binding. Thus, His86⇒Tyr86 replacement is likely to affect physical interaction of Rubisco with Rubisco activase. Although residue 86 is one of the most variable positions in the large subunit (up to 11 different amino acids across the 499 plant species; [26]), His86⇒Tyr86 replacement is very rare, considering that about 76% from 491 flowering species have His86, but only two species (<1%) have Tyr86 [15].

**Figure 2:**
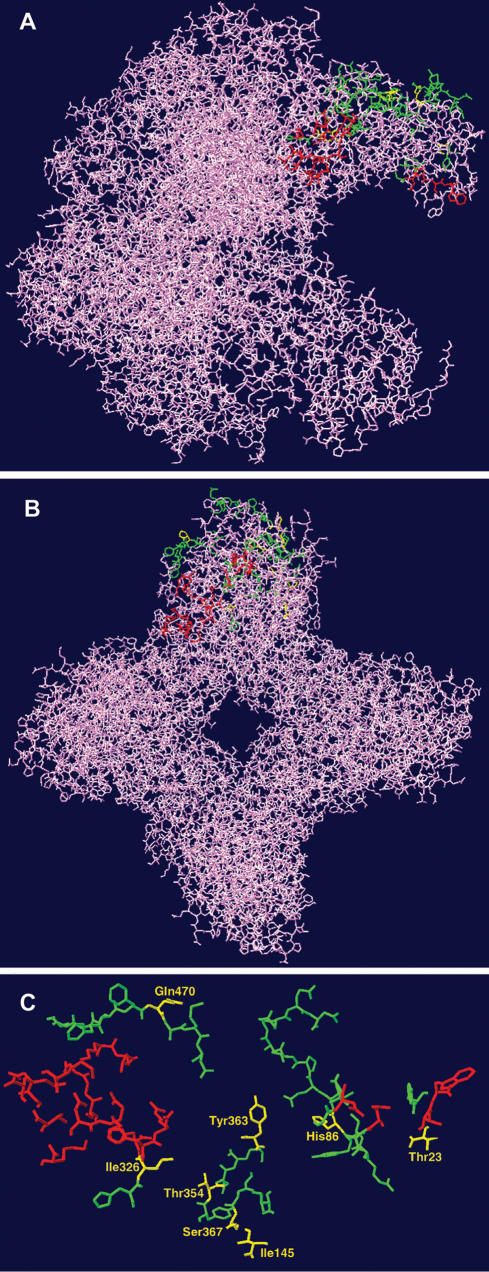
The structure of Rubisco enzyme in two projections (A, B) and (C) the residues that belong to active site (coloured red) and that are involved in interactions with Rubisco activase (coloured green; after [15], [24], [26], [27]). Eight residues replaced in *Schiedea* (positions 23, 86, 145, 326, 354, 363, 367, 470) are coloured yellow.

Furthermore, residue 86 is not the only one of *Schiedea rbcL* replacements that may be involved into Rubisco-Rubisco activase interactions. The critical residues for these interactions identified so far are immediately adjacent to the active site (Fig. 2, [27]), as well as residues in strand G (particularly strand 6)–strand H region and carboxyl terminus [15]. Based on published data [15], [27] and structural modelling we found that apart from residue 86 six other residues out of ten replacements in *Schiedea rbcL* could be involved in Rubisco-Rubisco activase interactions (residues 23 and 326 are close to the active site; residues 354, 363, 367 belong to strand G–strand H region and residue 470 is close to the carboxyl terminus). Six out of ten detected amino acid mutations while residing far from each other in the amino acid sequence appeared relatively close in the tertiary structure and could potentially influence each other: in the tertiary structure the average distance between the residues 86, 145, 326, 354, 363 and 367 is 15.1Å; between the residues 145, 326, 354 and 363 is 12.2Å; and between the residues 145, 354 and 363 is 9.2Å). The proximity of these replacements in the tertiary protein structure suggests that several mutations may have a cumulative effect that affects overall properties of Rubisco activase interaction region in *Schiedea*'s Rubisco (Fig. 2).

## Discussion

We demonstrated that the *rbcL* gene, encoding the large subunit of Rubisco enzyme, might have been under strong positive selection during recent adaptive radiation in Hawaiian *Schiedea*. Rubisco catalyzes the first step in net photosynthetic CO_2_ assimilation and photorespiratory carbon oxidation. The enzyme is subject to competitive inhibition by O_2_, inactivation by loss of carbamylation, and dead-end inhibition by RuBP, that makes Rubisco inefficient as a catalyst for the carboxylation of RuBP and limiting for photosynthesis and plant growth [15]. Thus, even small improvements in efficiency of this enzyme may provide significant physiological advantage.

In land plants Rubisco is composed of eight large subunits (LSUs) encoded by the chloroplast *rbcL* gene and eight small subunits (SSUs) encoded by a family of *rbcS* nuclear genes [28], [29]. By directed mutagenesis in *Rhodospirillum rubrum, Synechococcus, Chlamydomonas*, and tobacco it has been shown that even single mutations can positively or negatively change stability or substrate specificity of Rubisco [15], [30]. The most dramatic changes in Rubisco performance are inducted by replacements in the active site and in the regions providing interactions between LSUs and SSUs, and between Rubisco and Rubisco activase [15], [30].

Most amino acid replacements in *Schiedea rbcL* (residues 23, 86, 326, 354, 363, 367 and 470) reside in regions influencing interactions with Rubisco activase, a chaperone which promotes and maintains the catalytic activity of Rubisco [15], [27]. Rubisco activase plays a vital role in the response of photosynthesis to temperature [27], thus molecular adaptation of Rubisco-Rubisco activase interactions may have played an important role in adaptation of *Schiedea* species to dry sunny conditions. Furthermore, five of the replaced residues (86, 326, 354, 363 and 367) are close to each other (distances<20Å) in the Rubisco tertiary structure, suggesting possible cumulative effect. Sequencing and investigation of *Schiedea*'s Rubisco activase might be of considerable interest for future studies of possible coevolution of Rubisco and Rubisco activase in *Schiedea*.

The distribution of *rbcL* amino acid replacements in the *Schiedea* cpDNA phylogeny corroborates their possible functional importance. Non-synonymous mutations favored by positive selection are expected to be more common at the internal branches relative to terminal branches [31]. Indeed all amino acid replacements in *Schiedea* appeared in the internal branches (Fig. 1), a pattern significally different from ones of *rbcL* synonymous substitutions as well as from non-synonymous and synonymous mutations in other investigated cpDNA regions (Table 3).

The possible changes of Rubisco properties in *Schiedea* predicted from structural modeling match well with the observed difference in rates of photosynthesis [13] between “basal” and “advanced” species (roughly corresponding to clades I+II and III+IV, respectively) as well as with wide distribution of “advanced” *rbcL* haplotypes within *Schiedea*. The “basal” *Schiedea* species inhabit mesic or wet shady forests, while most species in the “advanced” clades (section *Schiedea*) colonised dry sunny habitats, such as coastal cliffs. Given the importance of Rubisco enzyme performance for plant growth and the significant effect of mutations affecting the contacts with Rubisco activase [15], [30], the His86⇒Tyr86 and other replacements in *Schiedea rbcL* may have played an important role in colonisation of dry habitats during recent adaptive radiation in *Schiedea*. Molecular adaptation in photosynthetic Rubisco enzyme represents the first known case of adaptation at the protein level during a recent adaptive radiation and reveals molecular bases of physiological and ecological evolution during rapid radiations in island endemics.

Positive selection on *rbcL* may possibly be the cause of fixation of two chloroplast haplotypes in virtually all species of *Schiedea* *sensu stricto* (clades III and IV on Fig. 1) and hence the main reason for cytonuclear discordance. This hypothesis is corroborated by the geographical pattern of *Schiedea* cpDNA haplotype distribution, where clades represent islands, rather than recognized *Schiedea* sections (Fig. 1).

Despite remarkable morphological and ecological divergence, natural interspecific hybrids have been found for many *Schiedea* species and the ability to cross-hybridize with each other has been shown for virtually all *Schiedea* species in green-house experiments [13]. However, strong geographical isolation between and within the islands makes interspecies contacts quite rare. Indeed, a previous DNA diversity study has demonstrated that isolation between the populations of *S. globosa* from different islands is much stronger than one between the populations from the same island [32]. Thus, it is quite likely that only genes under strong positive selection can spread across *Schiedea* species.

Positive selection on non-recombining chloroplast DNA is expected to lead to a spread of the selected chloroplast haplotype across several species, causing phylogenetic cytonuclear discordance. Cytonuclear discordance promoted by interspecific hybridization has been found in many adaptive radiations including Darwin's finches, African cichlids, Lake Baikal sculpins and Hawaiian silversword alliance (reviewed in [2]). Complete plastom and mitochondrion replacements via interspecific introgression have been documented for various plant and animal groups (reviewed in [33]). Most authors typically attribute the occurrence of introgression to demographic events and chance fixation, whereas relatively few suggest positive selection as a possible cause [34], [35]. The adaptive amino acid replacements in *Schiedea rbcL* occurred on the branches leading to the clades III and IV of the chloroplast gene tree. The spread of advantageous *rbcL* alleles across many *Schiedea* species inhabiting the same island (or a group of previously connected islands) argues in favor of positive selection as a main cause of cytonuclear discordance and suggests that sharing of adaptive mutations by several closely related species may be an important factor in adaptive evolution in small populations within confined geographical regions, such as oceanic islands or big lakes.

## Materials and Methods

### Isolation and sequencing of *Schiedea* genes

Morphology, ecology and origin of *Schiedea* species used in this study are presented in Table S1. Genomic DNA was isolated from fresh leaf material using magnetic beads-based Plant DNA Charge Switch Kit (Invitrogen) in accordance with manufacturer protocol. The primers used for amplification and sequencing are listed in Table S2. For PCR amplification of all regions except *trnS-trnG* we used BioMix Red (Bioline) with the following PCR conditions: one cycle of 95°C, 2 min, 55°C, 30 s, 72°C, 4 min followed by 36 cycles of 93°C, 30 s, 53°C, 30 s, 72°C, 3.5 min. For PCR amplification of *trnS-trnG* region we used Protocol 1 from [36]. The PCR products were extracted from the agarose gels using the Qiagen gel extraction kit. Sequencing was performed using ABI BigDye v3.1 system on an ABI3700 automated sequencing machine. Sequence chromatograms were checked and corrected, and the contigs were assembled and aligned using ProSeq3 software [37]. All polymorphic sites were checked against original sequence chromatograms and doubtful regions were resequenced; obtained sequences were compared with homologues from GenBank and ORFs integrity was confirmed for protein coding sequences; all indels were removed before further analyses. Novel sequences have been submitted to GenBank under accession numbers DQ907721-DQ907909.

### Statistical Tests for Positive Selection

The neighbor-joining trees for every investigated chloroplast region as well as for three concatenated datasets (noncoding regions, coding regions, all regions) were created using MEGA v3.1 [38]. The topologies of all obtained trees were similar and for further phylogenetic analyses of positive selection in *Schiedea*'s chloroplast protein coding genes we used the unrooted tree based on concatenated dataset of all regions.

We used the codeml program in the PAML v.3.14 [18] package to estimate the non-synonymous divergence (d*N*), synonymous divergence (d*S*), and their ratio (d*N*/d*S*) in model 0, that allows for a single d*N*/d*S* value throughout the whole phylogenetic tree. Further, codeml was used to perform likelihood ratio tests (LRTs) for rate heterogeneity and positive selection among amino acid sites. We applied models of codon evolution which allow for variation in d*N*/d*S* among codons but assume the same distribution in all branches of the phylogeny. We performed three LRTs for positive selection: M1a–M2a LRT, M7–M8 LRT and M8a–M8 LRT [21]–[23]. For all LRTs, the first model is a simplified version of the second, with fewer parameters, and is thus expected to provide a poorer fit to the data (lower maximum likelihood). The M1, M7 and M8a models are the null models without positive selection (no codons with d*N*/d*S*>1) and the M2 and M8 models are the alternative models with positive selection. The significance of the LRTs was calculated assuming that twice the difference in the log of maximum likelihood between the two models is distributed as a chi-square distribution with the degrees of freedom (df) given by the difference in the numbers of parameters in the two nested models. For both M1a–M2a and M7–M8 comparisons we used df = 2 [21], [22]. It was argued that for M8a–M8 comparisons the appropriate test would use a 50∶50 mixture of df = 0 and df = 1 [23], however we assumed df = 1 for this test, which is conservative [22].

To identify amino acid sites potentially under positive selection, the parameter estimates from M8 model were used to calculate the posterior probabilities that an amino acid belongs to a class with d*N*/d*S*>1 using the Bayes Empirical Bayes approaches implemented in PAML [25].

### Structural Analysis of Rubisco

We used spinach Rubisco protein structure [24] to infer the possible effect(s) of mutations at the residues identified as being under positive selection in *Schiedea*. The divergence between *rbcL*s of spinach and *Schiedea* at the amino acid level is between 3.2% and 4.4%, depending on the *Schiedea* species. Furthermore, the ancestral states of eight out of ten replacements found in *Schiedea rbcL* are identical to corresponding residues in spinach, making it appropriate to use protein structure obtained for spinach. Rubisco structural data for spinach (1RBO) were obtained from the RCB Protein Data Bank (http://www.rcsb.org/pdb). The solvent accessible surface areas for individual amino acids in the structure and the impact of single replacements on overall structural stability were analyzed using CUPSAT software [39; http://cupsat.uni-koeln.de]. The structural contacts for individual amino acids in the structure were analyzed using DeepView–Swiss-PdbViewer v. 3.7 [40; http://www.expasy.org/spdbv/] and STING Report [41; http://trantor.bioc.columbia.edu/SMS/].

## Supporting Information

Table S1 Habit, Habitat and Distribution of Investigated 27 Schiedea Species (after [13]).
(0.06 MB DOC)

Table S2 Investigated Chloroplast DNA Regions and Used Primers.
(0.04 MB DOC)
