# Supplementary material for: Molecular Adaptation during Adaptive Radiation in the Hawaiian Endemic Genus Schiedea
Source: PLoS One. 2006 Dec 20;1(1):e8. doi: 10.1371/journal.pone.0000008 (PMC1762304; doi:10.1371/journal.pone.0000008)
Supplement: Table S1 — Habit, Habitat and Distribution of Investigated 27 Schiedea Species (after [13]). (0.06 MB DOC) [file pone.0000008.s001.doc]

**Table S1.** Habit, Habitat and Distribution of Investigated 27 *Schiedea* Species (after ref. [13])

| Section, species | Habit^a^ | Habitat^b^ | Distribution^c^ |
| --- | --- | --- | --- |
| **Section** *Alphaschiedea* | |  |  |
| *S. helleri* | V | WF | K |
| *S. membranacea* | PH | MF | K |
| **Section** *Alsinidendron* | |  |  |
| *S. obovata* | S | MF | O |
| *S. trinervis* | S | WF | O |
| **Section** *Anestioschiedea* | |  |  |
| *S. apokremnos* | S | C | K |
| Section Mononeura |  |  |  |
| *S. jacobii* | PH | WF | EM |
| *S. kaalae* | PH | MF, WF | O |
| *S. kauaiensis* | SS | MF | K |
| *S. laui* | SS | WF | M |
| *S. nuttallii* | SS | MF | M (ext), O, WM (ext) |
| *S. pentandra* | V | MF | O |
| *S. perlmanii* | V | MS | K |
| *S. stellarioides* | SS | MF | K |
| **Section** *Nothoschiedea* | |  |  |
| *S. viscosa* | V | MF, WF | K |
| Section Polyneura |  |  |  |
| *S. verticillata* | PH | C | N |
| Section Schiedea |  |  |  |
| *S. adamantis* | S | DS | O |
| *S. globosa* | SS | C | EM, H, L (ext), M, O, WM |
| *S. haleakalensis* | S | C | EM |
| *S. hookeri* | SS | MF | EM (ext), O |
| *S. kealiae* | SS | DF | O |
| *S. ligustrina* | S | C, DF, DS | O |
| *S. lydgatei* | S | DS | M |
| *S. mannii* | S | Dry ridges in MF | O |
| *S. menziesii* | S | C, DS | L, WM |
| *S. salicaria* | S | C, DS | WM |
| *S. sarmentosa* | S | C, DF, DS | M |
| *S. spergulina* | S | C, DF, DS | K |

^a^ PH – perennial herb, S – shrub, SS – subshrub, V – vine.

^b^ C – dry cliffs, DF – dry forest, DS – dry shrubland, MF– diverse mesic forest, MS – mesic shrubland, WF – wet montane forest.

^c^ Abbreviations for separate Hawaiian islands: EM – East Maui, H – Hawai`i, K – Kaua`i, L – Lana`i, M – Moloka`i, N – Nihoa, O – O`ahu,, WM – West Maui, ext – extirpated.
