# Supplementary material for: Molecular Adaptation during Adaptive Radiation in the Hawaiian Endemic Genus Schiedea
Source: PLoS One. 2006 Dec 20;1(1):e8. doi: 10.1371/journal.pone.0000008 (PMC1762304; doi:10.1371/journal.pone.0000008)
Supplement: Table S2 — Investigated Chloroplast DNA Regions and Used Primers. (0.04 MB DOC) [file pone.0000008.s002.doc]

**Table S2. Investigated Chloroplast DNA Regions and Used Primers**

| Region | Primers |  | Primers reference |
| --- | --- | --- | --- |
| Protein coding genes (product, process) |  |  |  |
| *MatK* (maturase K, may be involved in splicing group II introns coding for tRNALys (UUU; Neuhaus & Link 1987) | MatK390F | CGATCTATTCATTCAATATTTC | [45] |
|  | MatK1130R | TCCGATAAATCGATCCAGACC | this study |
|  | MatK1000F | CAACCCATTGTCTTGACTTTATTGG | this study |
|  | PsbA2100R | GGTGCTGGTTATCCAGTTACAGAAG | this study |
| *PsbA* (photosystem II protein D1, photosynthesis) | Psb2040F | CAATTTTAGAGAGACGCGAAAGC | this study |
|  | Psb2900R | AGTAAACCAAATACCTACTACCGGC | this study |
| *RbcL* (ribulose-1,5-bisphosphate carboxylase/ | RbcL1F | ATGTCACCACAAACAGAAACTAAAGC | [46] |
| oxygenase large subunit, photosynthesis) | RbcL1460R | CTTTTAGTAAAAGATTGGGCCGAG | [46] |
| Noncoding regions |  |  |  |
| *psbA-trnK* intergenic spacer | MatK1000F | CAACCCATTGTCTTGACTTTATTGG | this study |
|  | PsbA2100R | GGTGCTGGTTATCCAGTTACAGAAG | this study |
| *tRNA-Leu* (*trnL*) gene, intron | trnL5’F | CGAAATCGGTAGACGCTACG | [47] |
|  | 3’trnLRD | GGGGATAGAGGGACTTGAAC | [47] |
| *trnL-trnF* intergenic spacer | 3’trnLRE | GGTTCAAGTCCCTCTATCCC | [47] |
|  | trnF | ATTTGAACTGGTGACACGAG | [47] |
| *trnS-trnG* intergenic spacer + *trnG* gene | trnS | AGATAGGGATTCGAACCCTCGGT | [36] |
|  | 3’trnG | GTAGCGGGAATCGAACCCGCATC | [36] |
